# Intra-peritoneal lavage of *Zingiber officinale* rhizome and its active constituent gingerol impede inflammation, angiogenesis, and fibrosis following post-operative peritoneal adhesion in male rats

**DOI:** 10.1016/j.jsps.2024.102092

**Published:** 2024-04-30

**Authors:** Roghayeh Yahyazadeh, Vafa Baradaran Rahimi, Seyed Ahmad Mohajeri, Milad Iranshahy, Maede Hasanpour, Vahid Reza Askari

**Affiliations:** a Department of Pharmacodynamics and Toxicology, School of Pharmacy, Mashhad University of Medical Sciences, Mashhad, Iran; b Pharmacological Research Center of Medicinal Plants, Mashhad University of Medical Sciences, Mashhad, Iran; c Department of Cardiovascular Diseases, Faculty of Medicine, Mashhad University of Medical Sciences, Mashhad, Iran; d Department of Pharmacognosy, School of Pharmacy, Mashhad University of Medical Sciences, Mashhad, Iran; e Biotechnology Research Center, Pharmaceutical Technology Institute, Mashhad University of Medical Sciences, Mashhad, Iran; f Applied Biomedical Research Center, Mashhad University of Medical Sciences, Mashhad, Iran

**Keywords:** Fibrosis, Inflammation, VEGF, Post-Surgical Adhesion, TGF-β, Ginger, Gingerol

## Abstract

Post-operative peritoneal adhesions (PA) are a common and important clinical problem. In this study, we focused on the ameliorative efficacy of ginger and gingerol compounds on surgical-induced peritoneal adhesion, and their strategies that disrupted the PA formation pathways to suppress their incidence. First, liquid chromatography-mass spectrometry (LC-MS) was established to separate and identify several chemical groups of ginger rhizome extract. In the next steps, male Wistar albino rats were randomly selected and divided into various groups, namely sham, control, ginger extract (0.6, 1.8, 5 %w/v), and gingerol (0.05, 0.1, 0.3, and 1 %w/v). Finally, we investigated the macroscopic parameters such as wound healing, body weight as well as spleen height and weight. In addition, visual peritoneal adhesion assessment was performed via Nair et al and Adhesion Scoring Scheme. Moreover, the microscopic parameters and biological assessment was performed via and immunoassays. The present findings revealed significant improvement in wound healing and reduction of the adhesion range, as Nair et al. and Adhesion Scoring Scheme scoring, in both the ginger and gingerol groups compared to the PA group (*P < 0.05*). Whereas, gingerol (0.3 % w/v) was able to increase the body weight in rats (*P < 0.0001*) at end stage of experiment. Also, inflammation, angiogenesis, and fibrosis were significantly decreased due to the downregulation of interleukin (IL)-6, tumor necrosis factor (TNF)-α, transforming growth factor (TGF)-β1, vascular endothelial growth factor (VEGF), respectively, in the ginger and gingerol groups compared to the PA group (*P < 0.05*). In contrast, the levels of IL-10 were increased in the ginger and gingerol groups compared to the control group (*P < 0.01*). Our results proved that ginger rhizome and gingerol, as novel therapeutic compounds, could be used to prevent PA for their beneficial anti-inflammatory as well as anti-fibrosis properties in clinical trials. However, further clinical studies are required to approve the effectiveness of ginger and gingerol.

## Graphical abstract

**Figure:**
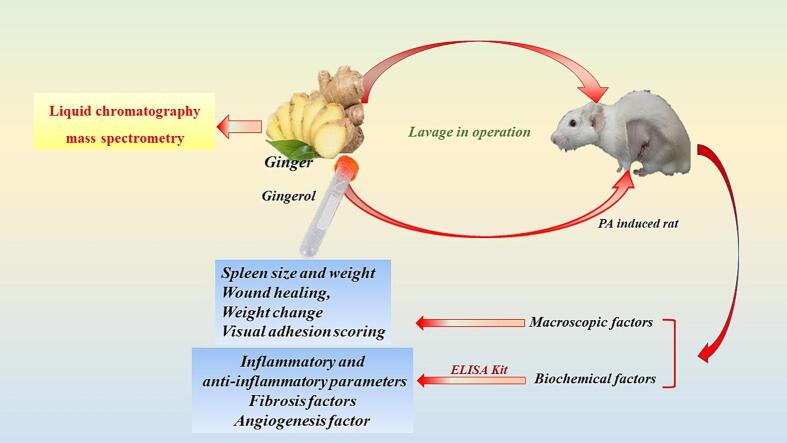

## 1 Introduction

Peritoneal adhesion (PA), an essential clinical issue following the operation/laparoscopic procedure, causes a wide range of disorders, such as bowel obstruction, abdominal pain, and female infertility (Makarchian et al., 2017). This problem may not only increase the reoperation rate and medical costs but also elongate the hospitalization time. A retrospective cohort study in Scotland indicates that 17.6 percent of surgical patients with peritoneal problems were may related to adhesions. Of these, 13.1 percent were bothered by adhesion, and 3.5 percent were significantly associated with PA (Krielen et al., 2020). Indeed, PA has a heavy economic burden on families and society (Krämer et al., 2021). The injured peritoneal mesothelial cells (PMC), as the main cells for adhesion formation (Wang et al., 2022), stimulate profibrotic cytokine expression (Fang et al., 2018) such as connective tissue growth factor (CTGF) and VEGF. Upregulation of these cytokines can cause inflammatory reactions and PA (Bekes et al., 2016). Peritoneal fluid, due to its constant interaction with the peritoneal surface, exhibits dynamic biochemical processes involved in adhesion progression. following peritoneal surgery, pro-inflammatory cytokines such as IL-1, IL-6, and TNF-α are liberated into the peritoneal cavity, potentially influencing adhesion formation and repair mechanisms (Hu et al., 2021). These cytokines likely contribute to adhesion formation through intricate pathways, IL-1 and TNF-α, both pro-inflammatory cytokines, play pivotal roles in the initial phases of wound healing and are produced by activated macrophages within peritoneal fluid (Zgheib et al., 2014). Conversely, IL-6 is synthesized by activated macrophages, with its production regulated by IL-1 throughout the inflammatory cascade. IL-1 and TNF-α are potent stimulators of IL-6 production (Tanaka et al., 2014). These cytokines are deemed significant due to their extensive interactions with the fibrinolytic pathway and their direct or indirect involvement in extracellular matrix (ECM) remodeling. Dysregulated control of ECM remodeling may underlie the formation of adhesions post-peritoneal injury (Sonbol, 2018).

Previous studies revealed that the injured PMC elevated the adhesion molecules, cytoskeleton restructured, loss of epithelial markers (Herrmann et al., 2020, Thakur et al., 2021), and marked mesenchymal cells. As a result, it leads to extensive extracellular matrix accumulation and epithelial-mesenchymal transition (Morishita et al., 2016, Strowitzki et al., 2017). Thus, anti-adhesion medications with material and life sciences have been interested in adhesion prevention, which can have advantages and disadvantages (Ten Broek et al., 2014, Yang Lili et al., 2019, Chandel et al., 2021).

The most effective strategy for PA prevention is avoiding surgery. as pharmaceutical approaches for PA treatment/prevention, Steroids, heparin, tissue-plasminogen activator (t-PA), and promethazine, have been used (Krämer et al., 2021). Scientists have suggested that the use of a combination of several extracellular mediators might exhibit a synergetic effect (Pugliese et al., 2018, Krämer et al., 2021). Although, in the in-vivo study, drug-releasing barrier systems were effective in PA prevention, but, no comprehensive data are available (Song et al., 2021). In this regard, their side effects during the healing process are an additional issue to be considered (Krämer et al., 2021). For instance, corticosteroids, as an immunosuppressant and poor wound healing, is a contraindication with anti-fibrinolytics using (Paladino et al., 2015). Accordingly, finding novel and effective compounds to inhibit PA seems necessary.

Medical herbs are used as beneficial substances in various fields, such as the food, pharmaceutical, and cosmetics industries (Dias et al., 2013, Yu et al., 2021). Given the World Health Organization (WHO) report, herbal medicines were commonly used by 80 % of the people in developing countries to get primary health care. This report indicates that plant-derived medicines consumption is increasing worldwide (Koraqi et al., 2022). *Zingiberaceae,* a family of herbal medicine, is being vastly investigated in the food industry and pharmacology (Balaji et al., 2022). Considering the importance of health and nutrition, medical plants as medicine, food, and pharmaceutical products are alternative compounds compared to synthetic products for their bioactive properties (Verma and Bisen, 2022). Ginger is the rhizome of *Zingiber officinale* Rosc. (*Zingiberaceae*) that is a local plant in China, India, and South East Asia. This plant is also known for its pharmacological properties, such as treating muscle spasms, joint pain, sore throats, fever, helminthiasis infection, dementia, acid reflux, and hypertension (Roudsari et al., 2021, Zhang et al., 2021). It has been shown that ginger is well-sustained without notable side effects and is composed of phenolic ingredients such as gingerols, shogaol, and paradol. Also, the investigation of these ingredients revealed that they have therapeutic properties for the innovation of modern medicine in managing disasters related to inflammation and oxidative stress. As a main ingredient of ginger, gingerol has excellent potential to be used as a medicine due to its anti-inflammatory, anti-oxidative, and anti-cancer CTGF properties (Yahyazadeh et al., 2023). Many studies have documented that inflammation and inflammation-related disorders are caused by the upregulation of toll-like receptors (TLRs), mitogen-activated protein kinase (MAPK), nuclear factor kappa-light-chain-enhancer of activated B cells (NF-κB), signal transducer and activator of transcription (STAT), mammalian target of rapamycin (mTOR), cyclooxygenase (COX)-2, and iNOS (Morvaridzadeh et al., 2020, Ozkur et al., 2022). Administration of ginger extract suppresses the expression of several inflammatory response genes and the above-mentioned (MAPK, NF-κB, STAT) pathways. Moreover, prostaglandin synthesis is suppressed following exposure to ginger via inhibition of COX-1 and COX-2 (Sani et al., 2014, Vishwakarma and Negi, 2020) Based on data from modern pharmacology and traditional knowledge, further studies regarding the effect of ginger and its ingredients are worthy for managing and treating PA in the future. However, the protective mechanism of ginger and gingerol on PA, related cytokines, biomarkers and fibrosis has not been investigated in detail. Therefore, the present study examined the ameliorative effect of ginger extract and gingerol on post-operative PA and peritoneal fibrosis using Wistar albino rats.

## 2 Materials and methods

### 2.1 Chemicals and kits

Ethanol was purchased from Sigma-Aldrich Chemical Co. (St. Louis, MO, USA). Normal saline (0.9 % w/v) was prepared from the Samen® pharmacy factory, Iran. Xylazine, acepromazine, and ketamine were prepared from Alfasen, Woerden, Holland. Also, enzyme-linked immunosorbent assay (ELISA) kits, including VEGF, IL-6, TGF-β, IL-10, and TNF-α, were obtained from MyBioSource, USA with catalog number respectively; MBS3015758, MBS8244673, MBS2701296, MBS2707969, MBS175904. Dichloromethane (DCM) was purchased from Caledon, Canada, for HPLC grade. In addition, deionized water and ethanol (96 %) were obtained from Abtin Co. (Iran), Kian Kaveh Azma Co. (Iran), and Taghtir Khorasan Co. (Iran), respectively. Furthermore, 6-gingerol was purchased from Golexir Pars ® Co., Iran, as an internal standard. Other chemicals and reagents were from Sigma-Aldrich Chemical Co. (St. Louis, MO, USA).

### 2.2 Plant preparation and extraction

Fresh ginger rhizomes were purchased from a local market in Mashhad. Mr. Joharchi, and Herbarium Research Institute faculty member of the Ferdowsi University of Mashhad, authenticated the rhizome as for *Zingiber officinale* Roscoe (herbarium No. E1260-FUMH). After washing, all were cut into small pieces. Afterward, the hydroethanolic extraction method was utilized to achieve the ginger extract in a suitable container at 37 °C for 72 h. Filtration was performed through filter paper, followed by evaporation in the incubator at 37 °C to obtain a solid powder. The obtained powder was stored in the freezer (−20 °C) for three years. The extract was prepared in normal saline with 5 % v/v Tween 80 (Ghadiri et al., 2021, Rakhshandeh et al., 2023, Yahyazadeh et al., 2023).

### 2.3 Identification of main ginger xanthones using liquid chromatography-mass spectrometry (LC-MS)

Liquid chromatography-mass spectrometry (LC-MS) analysis was carried out utilizing Agilent 1200 series liquid chromatography coupled with Agilent 6410 triple quadrupole Mass Spectrometer. Liquid chromatography was separated on an Agilent Eclipse plus C18 (2.1 × 100 mm × 3.5 μm) column. The flow rate was set to 0.4 mL/min, and the mobile phase consisted of (A) water + 0.1 % formic acid and (B) methanol + 0.1 % formic acid. The gradient programs were as follows: 0.0–1.0 min 10 % B, 1.0–40 min from 10 % to 100 % (B), 40.0–50.0 min 100 % (B), and 50.0–62.0 min from 100 % to 10 % (B). As a result, the mass spectra were acquired in the range of 100 to 1000 within a scan time of 62 min. A positive electrospray ionization (ESI) mode was applied for Mass Spectrometer. Mass feature extraction of acquired LC-MS data and maximum detection of peaks was done using the *MZmine* analysis software package, version 2.33.

### 2.4 Experimental animals

In this study, 66 male albino Wistar rats weighing 250 ± 40 g and 8–12 weeks old were purchased from the animal house of Mashhad University of Medical Sciences, Mashhad, Iran. All animals were familiarized for one week before the start of the study. Four rats were kept in each cage. All rats were housed under standard conditions with humidity of 50–60 % and atmospheric temperature (23 ± 2 °C) on a 12 h light–dark cycle with a standard diet/water *ad libitum*. Also, the details of their diet is brought in table 1. The study protocol was applied according to the National Institutes of Health (NIH) guidelines and accepted by the Local Animal Care Review Committee (20–10-2020, ethical approval code: IR.MUMS.MEDICAL.REC.1399.486).

### 2.5 Induction of peritoneal adhesion

General anesthesia was performed via an intraperitoneal (i.p.) injection of xylazine (10 mg/kg) and ketamine (100 mg/kg). After the rats' anesthesia, their abdominal skin was shaved and decontaminated with iodine solution and alcohol (Rahimi et al., 2017, Roohbakhsh et al., 2020, Jaafari et al., 2021). A three-centimeter incision was then created in the animal's abdomen to access the peritoneal cavity. For PA induction, the peritoneal scraping method was applied to the inside of the abdominal incision, which was gently abraded via soft sterilized gauze until the creation of an opaque presentation with fine petechia. Thereafter, the injured area in the peritoneal cavity was treated with 2 ml of gingerol/ginger extract solutions. After the intervention, the abdomen wall was stitched with a 4–0 poly-gelatin suture in the shortest time. After the surgical operation, rats were returned to their cages and monitored for seven days (Ghadiri et al., 2021, Rakhshandeh et al., 2023, Yahyazadeh et al., 2023). To prevent possible wound infection, a single dose of antibiotic (Cefazolin, 300 mg/kg) was also immediately injected intramuscularly after the operation (Stevens et al., 2014, Liang et al., 2018). It is noteworthy that PA-induction occurred on day zero at precise and consistent hours, and subsequent evaluations were conducted after seven days.

### 2.6 Experimental protocol

#### 2.6.1 Local treatment with ginger extract

The rats were randomly divided into five groups of 6 rats as follows:

Group I (Control): The rats with PA induction were not treated with ginger extract via lavage; Group II (Sham): The rats without PA induction were not treated with ginger extract via lavage; Group III (PA + 0.6 % w/v ginger extract): The rats not only underwent PA induction but also received 2 ml lavage containing sterilized distilled water containing 5 % tween 80 + 0.05 % w/v ginger extract; Group IV (PA + 1.8 % w/v ginger extract): The rats underwent PA induction and received 2 ml lavage containing sterilized distilled water containing 5 % tween 80 + 0.1 % w/v ginger extract; Group V (PA + 5 % w/v ginger extract): The rats underwent PA induction and received 2 ml lavage containing sterilized distilled water containing 5 % tween 80 + 0.3 % w/v ginger extract) (Ghadiri et al., 2021, Rakhshandeh et al., 2023, Yahyazadeh et al., 2023).

#### 2.6.2 Local treatment with gingerol

Local treatment with gingerol was conducted on 36 rats, which were divided into six groups (n = 6) as follows:

Group I (Control): The rats with PA induction were not treated with gingerol via lavage; Group II (Sham): The rats without PA induction were not given gingerol lavage; Group III (PA + 0.05 % w/v gingerol): The rats underwent PA induction followed by administration of 2 ml lavage containing sterilized distilled water containing 5 % tween 80 + 0.05 % w/v gingerol; Group IV (PA + 0.1 % w/v gingerol): The rats underwent PA induction and received 2 ml lavage containing sterilized distilled water containing 5 % tween 80 + 0.1 % w/v gingerol; Group V (PA + 0.3 % w/v gingerol): The rats not only underwent PA induction but also received 2 ml lavage containing sterilized distilled water containing 5 % tween 80 + 0.3 % w/v gingerol; Group Ⅵ (PA + 1 % w/v gingerol): The rats underwent PA induction and then received 2 ml lavage matrix (sterilized distilled water containing 5 % tween 80 + 1 % w/v gingerol) (Ghadiri et al., 2021, Rakhshandeh et al., 2023, Yahyazadeh et al., 2023).

### 2.7 Assessment of the macroscopic adhesion grade

On the seventh day after surgery, two adhesion grade scores were evaluated the PA grades following rat anesthesia and laparotomy, which was described by Nair et al. (1974) and Adhesion Scoring Scheme, Table 1, Table 2, respectively (Abbas et al., 2020, Jaafari et al., 2021, Kazemi et al., 2022). The adhesion was assessed in peritoneal cavity.

**Table 1:** The adhesion score classification based on Nair et al., (Nair et al., 1974, Ghadiri et al., 2021, Rakhshandeh et al., 2023, Yahyazadeh et al., 2023).

Grade	Description of Adhesive Bands
0	Complete absence of adhesions
1	A single band of adhesion between the viscera or from the viscera to the abdominal wall
2	Two bands, either between the viscera or from the viscera to the abdominal wall
3	More than two bands, between the viscera or the viscera up to the abdominal wall or whole intestines form a mass without being adherent to the abdominal wall
4	The viscera directly adherent to the abdominal wall, regardless of the number and extent of adhesive bands

**Table 2:** The adhesion score classification based on Adhesion Scoring Scheme (Askari et al., 2018, Ghadiri et al., 2021, Rakhshandeh et al., 2023, Yahyazadeh et al., 2023).

Grade	Description of Adhesive Bands
0	Absence of adhesions
1	A thin layer adhesion
2	More than a thin layer adhesion
3	Thick adhesive tissue attached to the surgical site
4	Thick adhesive tissue attached to different areas of the abdomen
5	Thick adhesive tissue containing blood vessels or too much adhesive tissue or organ adhesive tissue

### 2.8 Peritoneal lavage fluid collection and preparation

Sterilized phosphate-buffered saline (PBS) was used to prepare peritoneal lavage fluid after the rats' laparotomy. After washing the peritoneal cavity with PBS, the fluid was collected and then centrifuged at 3000 RPM for 5 min at 4 ℃. Finally, the obtained supernatant was utilized for further investigations.

### 2.9 Biochemical assays

In this study, TNF-α, IL-6, IL-10, VEGF, and TGF-β levels in the peritoneal fluid lavage were estimated using enzyme-linked immunosorbent assay (ELISA) kits (MyBioSource, USA) according to the manufacturer's instruction (Rahimi et al., 2017, Ghadiri et al., 2021, Rakhshandeh et al., 2023, Yahyazadeh et al., 2023).

### 2.10 Statistical analysis

The data were analyzed by GraphPad Prism (version 8.0.1) software according to the nature of parametric or nonparametric analysis. The parametric data were analyzed by one-way analysis of variance (ANOVA) or Brown-Forsythe multiple comparison tests followed by a two-stage step-up method of Benjamini, Krieger, and Yekutieli post-test. Also, the nonparametric data, such as adhesion scores, were analyzed by the Kruskal-Wallis’s test and the two-stage step-up method of Benjamini, Krieger, and Yekutieli's multiple comparison post hoc test. P values (P) ≤ 0.01 were considered statistically significant, and data were also expressed as means ± SD or median ± interquartile range (IQR) (Ghadiri et al., 2021, Rakhshandeh et al., 2023, Yahyazadeh et al., 2023).

## 3 Results

### 3.1 LC-MS analysis of *Zingiber officinale* extract

**Fig. 1:**
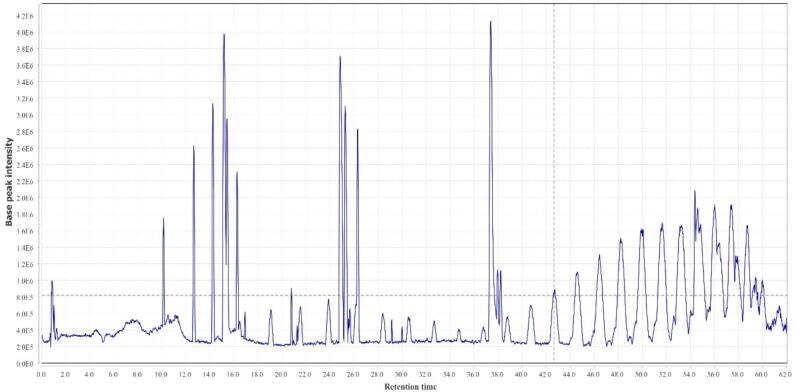
The total ion chromatogram of *Zingiber officinale* extract.

**Fig. 2:**
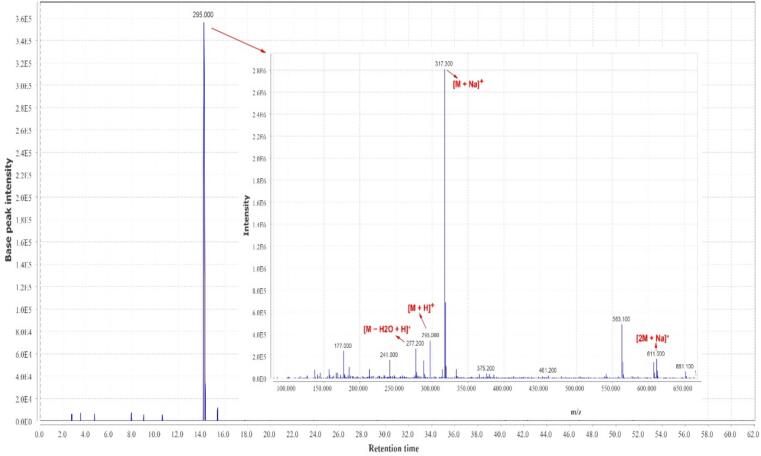
Chromatogram of 6-gingerol and corresponding mass adduct, [M + 1], at *m*/*z* 295.0.

**Fig. 3:**
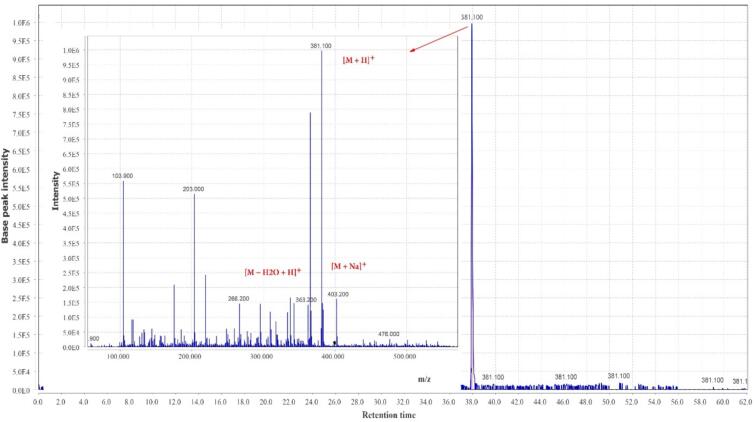
Chromatogram of diacetoxy-6-gingerdiol and corresponding mass adduct, [M + 1], at *m*/*z* 381.1.

**Fig. 4:**
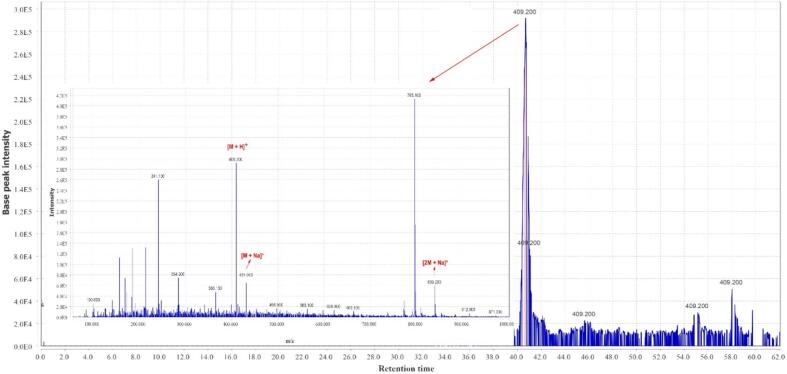
Chromatogram of diacetoxy-8-gingerdiol and corresponding mass adduct, [M + 1], at *m*/*z* 409.2.

A total of 22 compounds were identified in the hydro-ethanol extract of *Zingiber officinale*, which have shown in Table 3 at several groups of compounds, including the gingerols, methylgingerols, gingerol acetates, shogaols, paradols, gingerdiols, mono- and diacetyl gingerdiols, and dehydrogingerdiones. The total ion chromatogram of *Zingiber officinale* extract is shown in Fig. 1. The MS spectral data were compared with the reported compounds in some previous literature (Kiyama, 2020, Unuofin et al., 2021). Examples of extracted ion chromatograms from the total ion chromatogram and its related mass are given in Fig. 2, Fig. 3, Fig. 4. Most of the compounds detected in the *Zingiber officinale* extracts have been previously reported in ginger.

**Table 3:** Peak assignment of metabolites in hydro-ethanol extract of ginger using LC–MS in positive mode.

**No**	**Retention time (min)**	**Compounds**	**Formula**	**Ions**	**Exptl**	**Ref.**
1	13.7	4-Gingerol	C15H22O4	[M + H]^+^	267.1	(Jiang et al., 2005)
				[M + Na]^+^	289.2	
				[M − H_2_O + H]^+^	249.2	
2	14.2	6-Gingerol	C17H26O4	[M + H]^+^	295.0	(Jiang et al., 2005, Tao et al., 2009)
				[M + Na]^+^	317.3	
				[2 M + Na]^+^	611.2	
				[M − H_2_O + H]^+^	277.2	
3	18.4	Dehydro-6-gingerol	C17H24O4	[M + H]^+^	293.1	(Tao et al., 2009)
				[M + Na]^+^	315.3	
				[M − H_2_O + H]^+^	275.1	
4	41.2	Methyl −6-Gingerol	C18H28O4	[M + H]^+^	309.3	(Jiang et al., 2005)
				[M + Na]^+^	331.2	
				[M − H_2_O + H]^+^	291.1	
5	29.1	8-Gingerol	C19H30O4	[M + H]^+^	323.2	(Jiang et al., 2005)
				[M + Na]^+^	345.3	
				[M − H_2_O + H]^+^	305.2	
6	26.2	6-Shogaol	C17H24O3	[M + H]^+^	277.2	(Park and Jung, 2012)
				[M + Na]^+^	299.1	
				[M + K]^+^	375.1	
				[M − H_2_O + H]^+^	259.1	
7	25.7	Dehydro-8-gingerol	C19H28O4	[M + H]^+^	321.1	(Tao et al., 2009, Park and Jung, 2012)
				[M + Na]^+^	343.3	
				[M − H_2_O + H]^+^	303.2	
8	31.3	Methyl −8-gingerol	C20H32O4	[M + Na]^+^	359.3	(Park and Jung, 2012)
				[M + K]^+^	375.2	
				[M − H_2_O + H]^+^	319.1	
9	22.1	Dehydro-6-gingerdione	C17H22O4	[M + H]^+^	291.2	(Park and Jung, 2012)
				[M + Na]^+^	313.2	
				[2 M + Na]^+^	603.8	
10	15.4	10-Gingerol	C21H34O4	[M + H]^+^	351.3	(Tao et al., 2009)
				[2 M + H]^+^	701.1	
				[M + Na]^+^	373.2	
				[M − H_2_O + H]^+^	333.1	
11	29.8	8-Shogaol	C19H28O3	[M + H]^+^	305.1	(Tao et al., 2009, Park and Jung, 2012)
				[M + Na]^+^	327.2	
				[M + K]^+^	343.1	
12	40.3	Dehydro-8-gingerdione	C19H26O5	[M + H]^+^	319.3	(Park and Jung, 2012)
				[M + Na]^+^	341.3	
13	52.5	12-Gingerol	C23H38O4	[M + H]^+^	379.3	(Tao et al., 2009)
				[M + Na]^+^	401.2	
				[M − H_2_O + H]^+^	361.3	
14	40.5	10-Shogaol	C_21_H_32_O_3_	[M + H]^+^	333.3	(Tao et al., 2009)
				[M + Na]^+^	355.3	
15	54.2	Dehydro-10-gingerdione	C21H30O4	[M + H]^+^	347.4	(Park and Jung, 2012)
				[M + Na]^+^	369.3	
16	55.8	Dehydro-12-gingerdione	C23H34O4	[M + H]^+^	375.2	(Park and Jung, 2012)
				[M + Na]^+^	397.2	
17	58.4	Dehydro-14-gingerdione	C25H38O4	[M + H]^+^	403.2	(Park and Jung, 2012)
				[M + Na]^+^	425.2	
18	45.1	Methyl 3-acetoxy-6-gingerdiol	C20H32O5	[M + H]^+^	353.3	(Jiang et al., 2005, Asamenew et al., 2019)
				[M + Na]^+^	375.0	
19	37.9	6-Paradol	C17 H26 O3	[M + H]^+^	279.1	(Jiang et al., 2005)
				[M + Na]^+^	301.2	
20	38.0	Diacetoxy-6-gingerdiol	C21H32O6	[M + H]^+^	381.1	(Jiang et al., 2005)
				[M + Na]^+^	403.2	
				[M − H_2_O + H]^+^	363.2	
21	40.7	Diacetoxy-8-gingerdiol	C23H36O6	[M + H]^+^	409.2	(Jiang et al., 2005)
				[M + Na]^+^	431.9	
				[2 M + Na]^+^	839.2

### 3.2 Evaluation of ginger extract effects on macroscopic parameters

In this study, seven days after the peritoneal adhesion induction, all animals were evaluated for macroscopic parameters such as spleen size and weight, wound healing, and weight change. The severity of adhesion formation was then examined via suitable scoring (Nair et al. and Adhesion Scoring Scheme). Local administration of ginger extract showed a significant difference in wound length in the 0.6 (*P < 0.05*) and 1.8 w/v % (*P < 0.0001*) groups compared to the control group. On the contrary, there were no significant differences between the ginger extract groups and the control group in size and weight of the spleen and weight change (Table 4).

**Table 4:** Evaluation of ginger extract on macroscopic parameters. Data were presented as Means ± SD.

	**Day**	**Sham**	**Control**	**0.6 % w/v**	**1.8 % w/v**	**5 %w/v**	**P-value**
**Weight**	0	250.5 ± 9.5	263.3 ± 5.7	250.7 ± 10.3	258.3 ± 6.7	259.8 ± 12.2	NO
	8	255.3 ± 8.7	264.7 ± 5.3	254.5 ± 8.5	263.3 ± 7.7	264.7 ± 10.3	NO
**Spleen size**	8	39.83 ± 1.722	39.83 ± 1.602	39.83 ± 1.169	39 ± 1.265	41.33 ± 2.422	NO
**Spleen weight**	8	929.5 ± 119.2	917 ± 124.8	975 ± 140.7	802.5 ± 34.60	1060 ± 254	NO
**Wound healing**	0	26 ± 0.812	26.50 ± 0.761	26.17 ± 1.066	26.83 ± 0.687	26.50 ± 1.25	NO
	8	19.33 ± 0.942	19.17 ± 1.06	14 ± 1.91***	6 ± 4.24***	16.33 ± 2.284	*** *P < 0.0005*

**Fig. 5:**
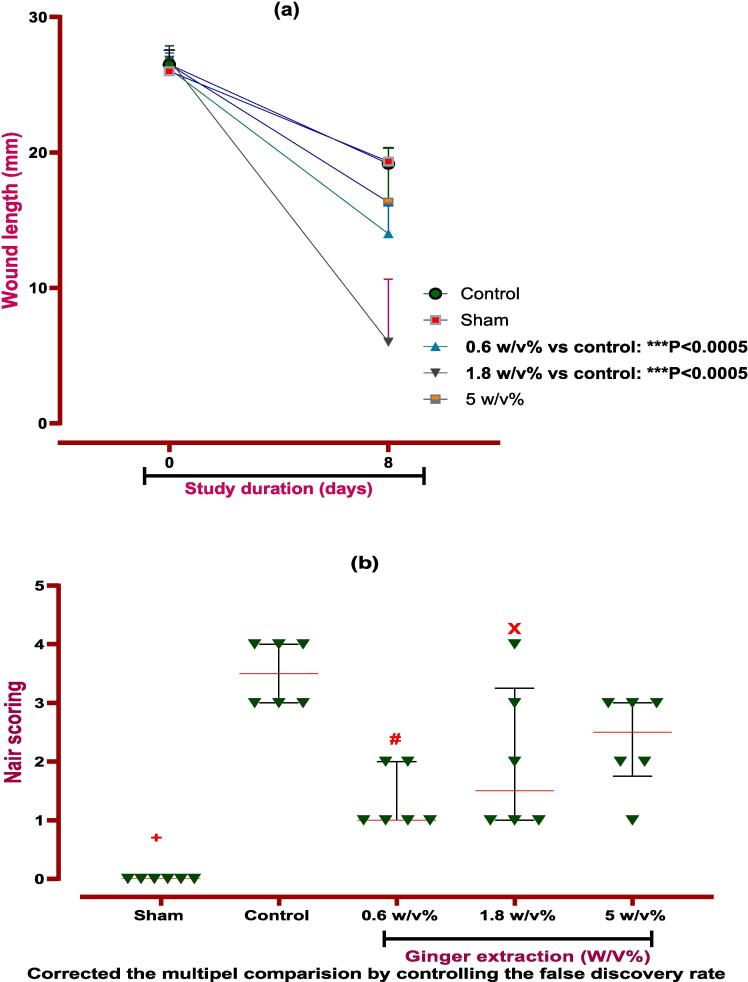

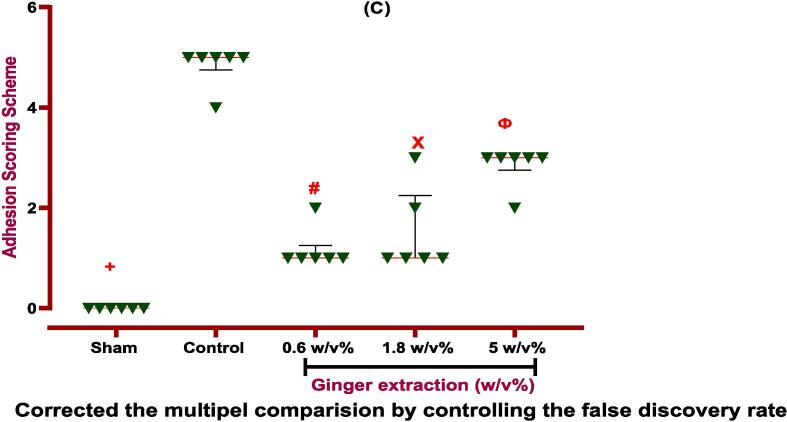
**Evaluation of ginger extract effect on PA macroscopic parameter. (a) The effects of various doses of ginger extract on wound healing**. **Evaluation of ginger extract on PA via (b) Nair et al. scoring.**+: Control vs group of sham % w/v; ***P < 0.0001, #: Control vs group of 0.6 % w/v; **P < 0.01,***×***: Control vs group of 1.8 % w/v; **P < 0.01. **(c) Adhesion scheme scoring:**+: Control vs group of sham % w/v; ***P < 0.0001, #: Control vs group of 0.6 % w/v; ***P < 0.0001,***×***: Control vs group of 1.8 % w/v; **P < 0.01, Ф: Control vs group of 5 % w/v; *P < 0.05. All data were represented as Means ± SD, except for adhesion scores indicating by median ± IQR (n = 6 for each group). Repeated-measurement two-way ANOVA with Two-stage step-up method of Benjamini, Krieger and Yekutieli multiple comparison post hoc tests were used for data analyzing.

**Fig. 6:**
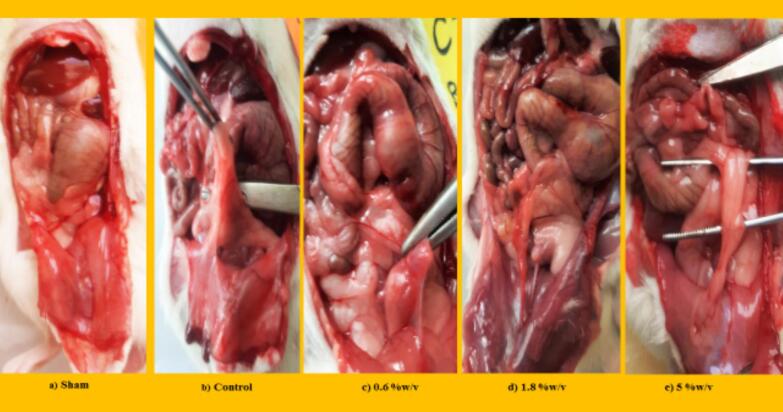
Evaluation of ginger extract effect in various doses on PA.

Finally, the evaluation of incision sites in rats proved that ginger extract is able to improve the abdominal wound via local administration without infection or other complications. So, the visual assessment of Nair et al. and adhesion scheme scoring revealed that elevated PA score in the control group was reduced via ginger extract. According to the Nair et al. scoring, ginger extract with 0.6 and 1.8 % w/v concentrations is able to improve the wound. But, the concentration of 0.6 % w/v is more effective than 1.8 % w/v ginger extract on wound healing. Unlike Nair et al. scoring, in adhesion scheme scoring, all three doses (0.6, 1.8, and 5 % w/v) cause better wound healing compared to the control group. It is worth mentioning the dose of 0.6 % w/v ginger extract is most effective (*P ≤ 0.0001*) on PA, as explained and brought in Fig. 5, Fig. 6. It is worth mentioning that ginger extract in i.p. rout could not affect on body weight and spleen height and weight after seven days in PA-induced rats.

### 3.3 Evaluation of gingerol effects on macroscopic parameters

**Fig. 7:**
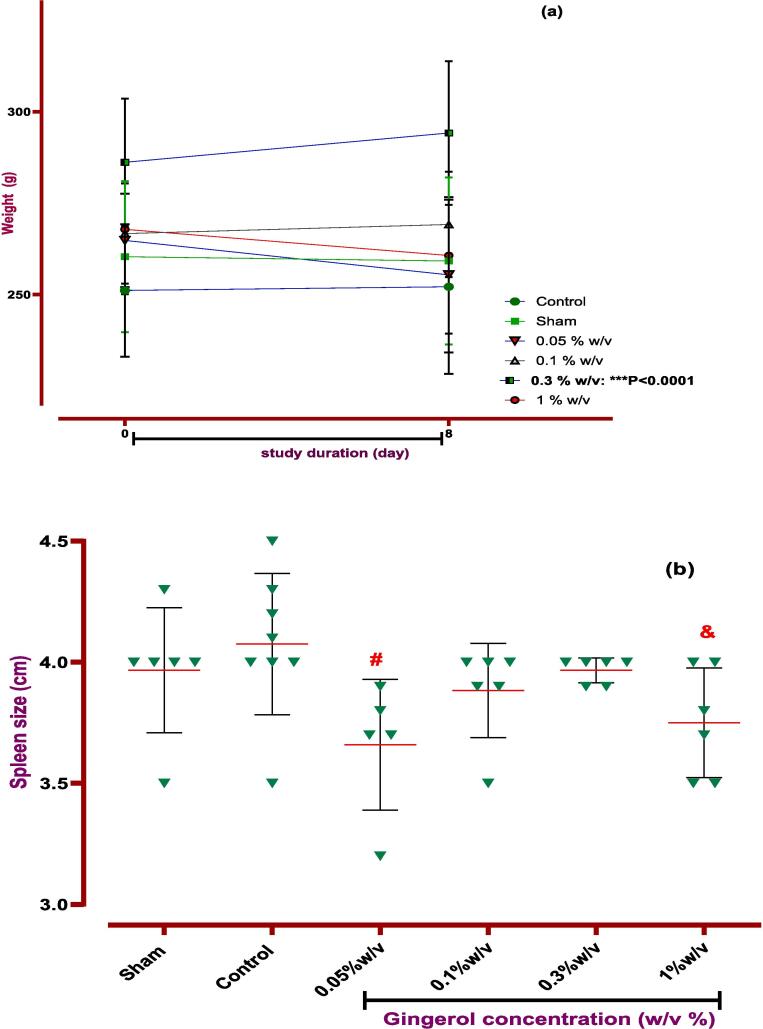

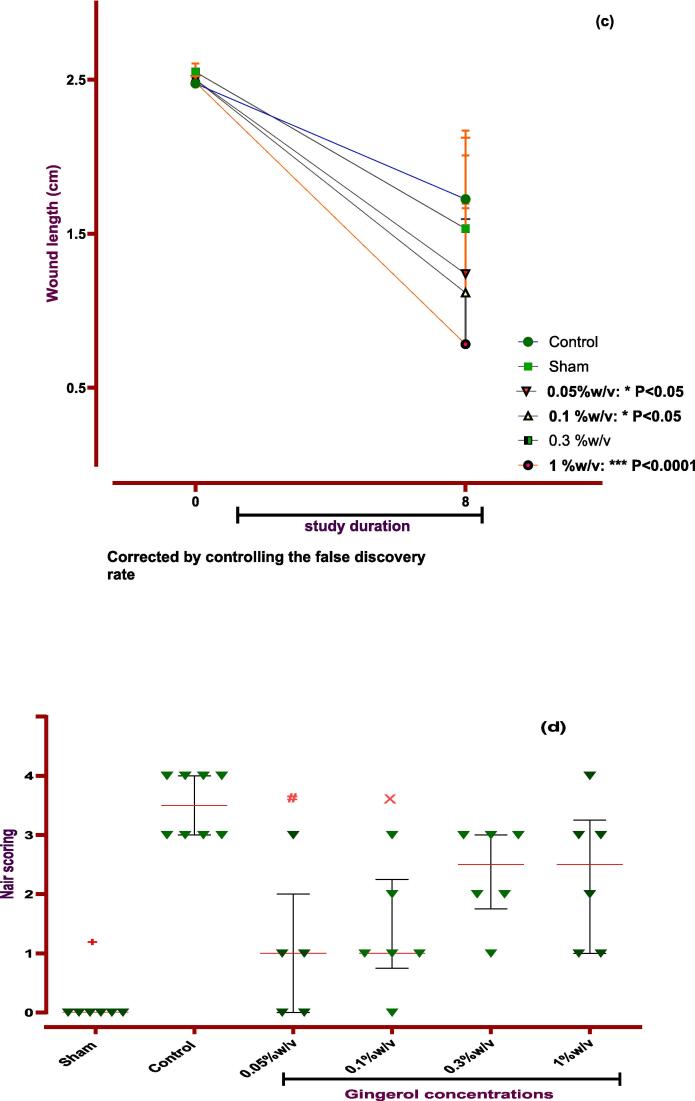

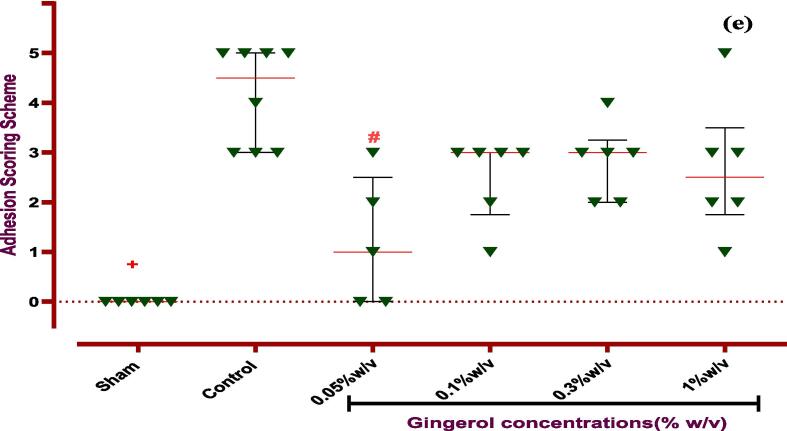
**Evaluation of gingerol effect on PA macroscopic parameter. The effects of various doses of gingerol on (a) weight changing, (b) spleen size:**#: Control vs group of 0.05 % w/v; *P < 0.05, &: Control vs group of 1 % w/v; *P < 0.05,***(c) wound healing, (d) Nair et al. scoring***+: Control vs group of sham % w/v; ***P < 0.0001, #: Control vs group of 0.05 % w/v; *P < 0.05, ×: Control vs group of 0.1 % w/v; *P < 0.05.**(e) Adhesion scoring scheme:**+: Control vs group of sham % w/v; ***P < 0.0001, #: Control vs group of 0.05 % w/v; **P < 0.01. All data were represented as Means ± SD, except for adhesion scores indicating by median ± IQR (n = 6 for each group). Repeated-measurement two-way ANOVA with Two-stage step-up method of Benjamini, Krieger and Yekutieli multiple comparison post hoc tests were used for data analyzing.

**Fig. 8:**
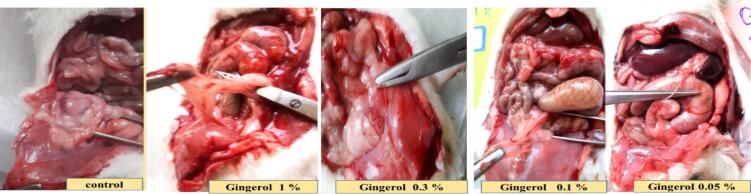
Evaluation of gingerol effect in various doses on PA.

At the end of the investigation, the rat's incisions were primarily assessed without infection or related complications. This paper explored macroscopic parameters such as spleen size and weight, wound healing, and weight change to assess gingerol properties. So, examination revealed that gingerol with 0.05 and 1 % w/v concentration could significantly decrease the spleen size at the end of the study period (*P < 0.05*). These parameters were measured at zero and the 8th day to evaluate wound healing and weight change. The results show that local administration of gingerol (0.05, 0.1, and 1 % w/v) in the peritoneal cavity significantly improved wound healing after seven days in rats (*P < 0.05* and *P < 0.0001*). Nevertheless, only 0.3 % w/v was able to increase the body weight in rats (*P < 0.0001*, Fig. 7, Fig. 8 and Table 5). In addition, the investigation showed that gingerol had no significant influence on spleen weight compared to the control group (Table 5).

**Table 5:** Evaluation of gingerol on macroscopic parameters.

	**Day**	**Sham**	**Control**	**0.05 % w/v**	**0.1 % w/v**	**0.3 % w/v**	**1 %w/v**	**P-value**
**Weight**	0	260.3 ± 30.7	251.1 ± 24.9	264.8 ± 15.2	266.7 ± 23.3	286.2 ± 12.8	267.8 ± 32.2	NO
	8	259.2 ± 34.8	252.1 ± 31.9	255.4 ± 24.6	269.2 ± 19.8	294.2 ± 17.8***	260.7 ± 30.3	***P < 0.001
**Spleen size**	8	3.967 ± 0.258	4.075 ± 0.2915	3.660 ± 0.2702*	3.883 ± 0.194	3.967 ± 0.0516	3.750 ± 0.2258*	*P < 0.05
**Spleen weight**	8	960.7 ± 171.6	864.0 ± 173.1	864.0 ± 185.3	751.7 ± 120.2	996 ± 147.1	933.3 ± 386.3	NO
**Wound healing**	0	25.50 ± 5	24.75 ± 0.866	25 ± 0.632	25 ± 0.44	25.5 ± 0.25	24.83 ± 0.484	NO
	8	15.33 ± 1.45	17.25 ± 3.64	12.4 ± 63.46*	11.2 ± 25.14*	15.3 ± 18.89	7.83 ± 7.425***	*P < 0.05 ***P < 0.001

Data were presented as Means ± SD.

Also, the Nair et al. and Adhesion scheme scoring was used to assess the gingerol effect on induced post-operative PA rate. The examination proved that the PA rate was significantly increased in the control group against the sham group (*P < 0.0001* for both scoring systems). Although, according to Nair et al. scoring, 0.05 and 0.1 % w/v of gingerol prominently decreased the PA scoring rate compared to the control group (*P < 0.05*). But, according to Adhesion scheme scoring, only 0.05 % w/v gingerol group significantly affected PA compared to the control group (*P < 0.005*, Fig. 7, Fig. 8, and Table 5).

### 3.4 Effects of ginger extract on the cytokine’s expression levels

#### 3.4.1 Effects of ginger on inflammatory and anti-inflammatory factors

**Fig. 9:**
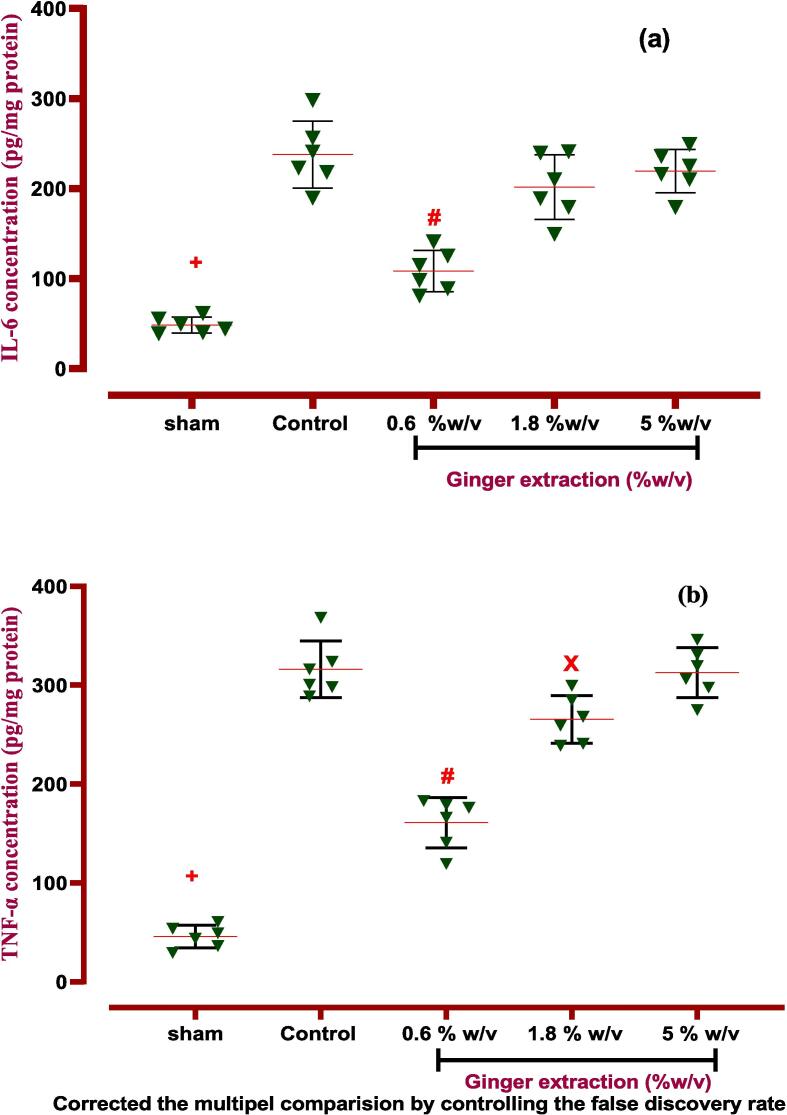

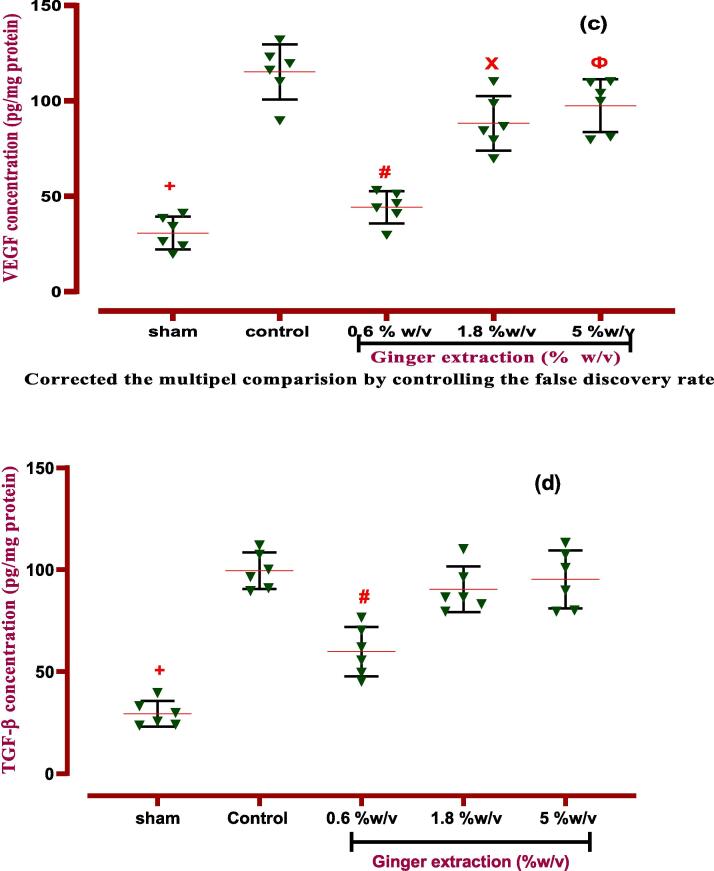
**The effects of ginger extract on PA in the peritoneal lavage levels of (a) IL-6:**+: Control vs group of sham % w/v; ***p < 0.0001, #: Control vs group of 0.6 % w/v; ***p < 0.0001***,* (B) TNF-α:**+: Control vs group of sham % w/v; ***p < 0.0001, #: Control vs group of 0.6 % w/v; ***p < 0.0001, ×: Control vs group of 1.8 % w/v; ***p < 0.001,**(c) VEGF**: +: Control vs group of sham % w/v; ***p < 0.0001, #: Control vs group of 0.6 % w/v; ***p < 0.0001, ×: Control vs group of 1.8 % w/v; **p < 0.01, Ф: Control vs group of 5 % w/v; *p < 0.05***,* (d) TGF-β:**+: Control vs group of sham % w/v; ***p < 0.0001, #: Control vs group of 0.6 % w/v; ***p < 0.0001. Data were possessed as Means ± SD (n = 6). Ordinary one-way ANOVA and Brown-Forsythe one-way analysis of variance (ANOVA) was used via Two-stage step-up method of Benjamini, Krieger and Yekutieli multiple comparisons post hoc test for parametric data. ∗ Compared the treated group with the control group; *: P < 0.05, **: P < 0.01 and ***: P < 0.001.

Our study revealed that the inflammatory biomarkers (IL-6, TNF-α) were significantly elevated in PA-induced rats compared to the normal group (*P < 0.001*). But, ginger extract (0.6 % w/v) was able to downregulate the expression of IL-6 in fluid lavage of the peritoneal cavity (*P < 0.0001*). Whereas, the extract at two doses of 0.6 and 1.8 % w/v significantly decreased the expression of TNF-α against the control group with p values of *P < 0.0001* and *P < 0.05*, respectively (Fig. 9, Table 6). In addition, our experiments showed that ginger extract had not any potential effect on IL-10 concentrations.

**Table 6:** ANOVA table for all immunological and biochemical parametric data with ginger extract.

	groups	F* (DFn, DFd)	Means ± SD	P-value	Type of ANOVA test
IL-6		1.968 (4, 25)		*p < 0.001*	Ordinary one-way ANOVA
	Sham		48.34 ± 8.934	*p < 0.0001*	
	Control		237.8 ± 37.18	−	
	0.6 %w/v		108.4 ± 22.88	*p < 0.0001*	
	1.8 %w/v		201.6 ± 35.86	Not	
	5 %w/v		219.4 ± 24.06	Not	
IL-10		9.972 (4.000,13.77)		*P < 0.001*	Brown-Forsythe- Dunnett's-T3
	Sham		20.03 ± 5.075	*P < 0.001*	
	Control		51.41 ± 6.99	−	
	0.6 %w/v		62.32 ± 20.33	Not	
	1.8 %w/v		5.28 ± 11.03	Not	
	5 %w/v		52.18 ± 12.52	Not	
TNF-α		0.5660 (4, 25)		*P < 0.001*	Ordinary one-way ANOVA
	Sham		45.79 ± 11.53	*P < 0.0001*	
	Control		316.2 ± 28.59	−	
	0.6 %w/v		160.9 ± 25.28	*P < 0.001*	
	1.8 %w/v		265.4 ± 24.3	Not	
	5 %w/v		312.8 ± 25.13	Not	
TGF-β1		1.285 (4, 25)		*P < 0.0001*	Ordinary one-way ANOVA
	Sham		2.38 ± 6.239	*P < 0.0001*	
	Control		99.51 ± 8.949	−	
	0.6 %w/v		59.92 ± 12.09	*P < 0.0001*	
	1.8 %w/v		90.45 ± 11.18	Not	
	5 %w/v		95.28 ± 14.23	Not	
VEGF		0.3943 (4, 25)		*P < 0.001*	Ordinary one-way ANOVA
	Sham		30.67 ± 8.585	*P < 0.0001*	
	Control		115.1 ± 14.43	−	
	0.6 %w/v		44.23 ± 8.433	*P < 0.0001*	
	1.8 %w/v		88.18 ± 14.32	*P < 0.005*	
	5 %w/v		97.42 ± 13.76	*P < 0.05*	

Dfn: the degree of freedom for the numerator of the F ratio, DFd: degrees of freedom denominator, IL: Interleukin, TNF-α: tumor necrosis factor-alpha, VEGF: vascular endothelial growth factor, TGF-β1: transforming growth factor beta 1. *P*-value is shown compared with control groups.

#### 3.4.2 Effects of ginger on angiogenesis and fibrosis factors

We also investigated the effects of ginger extract on angiogenesis and fibrosis biomarkers levels. Induction of PA caused an upregulation of VEGF and TGF-β expression range in the control group against to normal group in rats (*P < 0.0001*). As a result, VEGF expression level, as an angiogenesis factor, downregulated via all three doses of ginger extract (0.6, 1.8, and 5 % w/v, *P < 0.0001, P < 0.005*, and *P < 0.05*, Table 6, Fig. 9). Nevertheless, the ginger extract at the dose of 0.6 % w/v significantly diminished the levels of TGF-β1 compared to the control group (*P < 0.0001*).

### 3.5 Effects of gingerol on cytokines expression level

#### 3.5.1 Effects of gingerol on inflammatory and anti-inflammatory factors

**Fig. 10:**
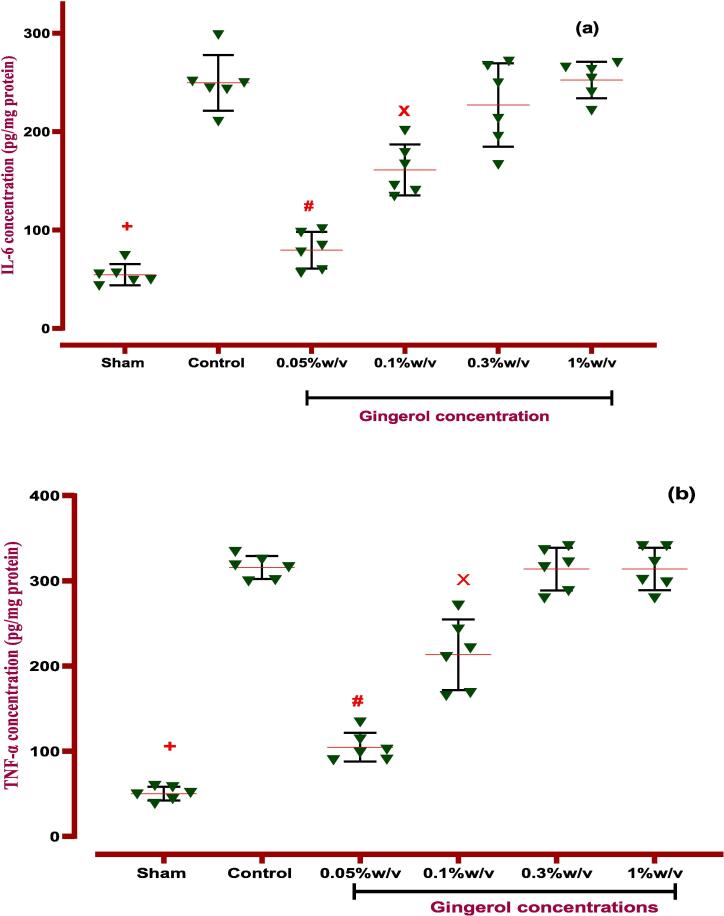

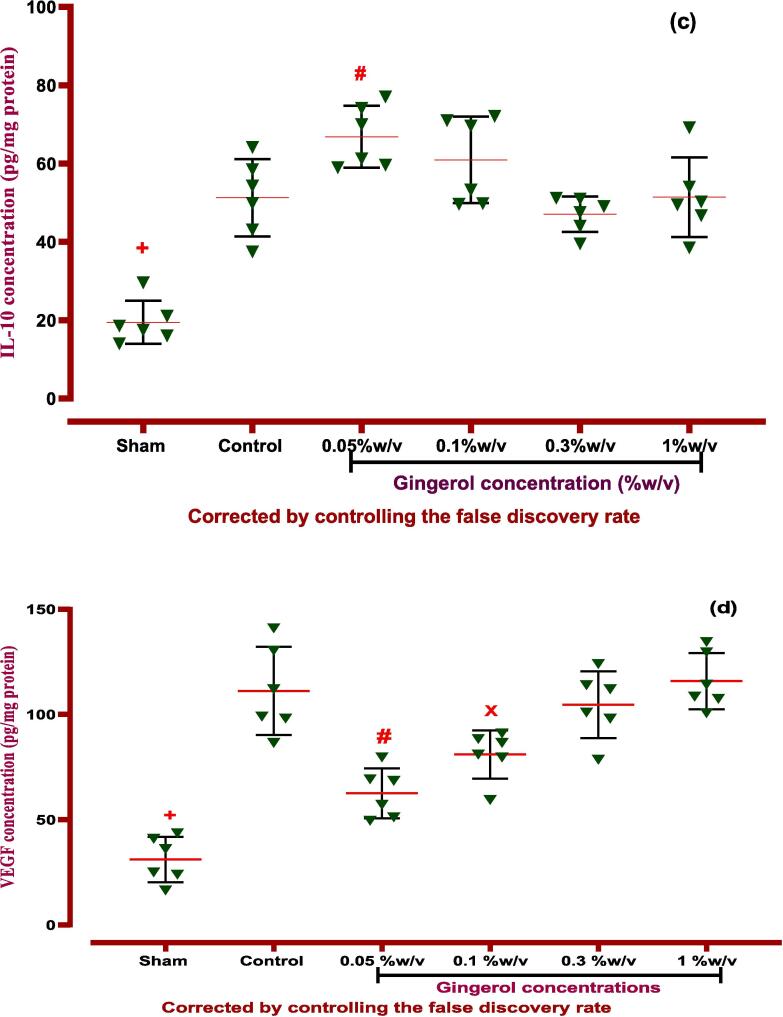

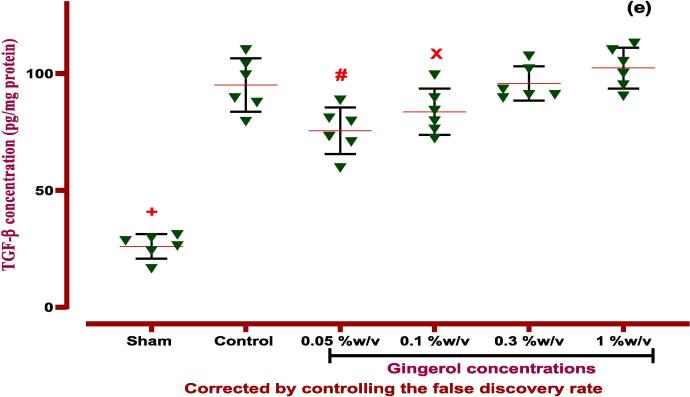
**The effects of gingerol on PA in the peritoneal lavage levels of (a) IL-6*:***+: Control vs group of sham % w/v; ***p < 0.0001, #: Control vs group of 0.05 % w/v; ***p < 0.0001, ×: Control vs group of 0.1 % w/v; **p < 0.01,**(b) TNF-α:**+: Control vs group of sham % w/v; ***p < 0.0001, #: Control vs group of 0.05 % w/v; ***p < 0.0001, ×: Control vs group of 0.1 % w/v; **p < 0.01,**(c) IL-10:**+: Control vs group of sham % w/v; ***p < 0.0001, #: Control vs group of 0.05 % w/v; **p < 0.01,**(d) VEGF*:***+: Control vs group of sham % w/v; ***p < 0.0001, #: Control vs group of 0.05 % w/v; ***p < 0.0001,***×***: Control vs group of 0.1 % w/v; ***p < 0.001,**(e) TGF-β:**+: Control vs group of sham % w/v; ***p < 0.0001, #: Control vs group of 0.05 % w/v; **p < 0.01,***×***: Control vs group of 0.1 % w/v; *p < 0.05. Data were possessed as Means ± SD (n = 6). Ordinary one-way ANOVA and Brown-Forsythe one-way analysis of variance (ANOVA) were used via Two-stage step-up method of Benjamini, Krieger and Yekutieli multiple comparisons post hoc test for parametric data. ∗ Compared the treated group with the control group; *: P < 0.05, **: P < 0.01 and ***: P < 0.001.

Following PA induction, all inflammatory cytokines, such as IL-6 and TNF-α, were elevated significantly compared to the normal group (sham) (*P < 0.0001*). Therefore, local treatment of gingerol (0.05 and 0.1 %w/v) could downregulate the mentioned inflammatory cytokines in peritoneal cavity lavage fluid (*P < 0.0001, P < 0.01* for both IL-6 and TNF-α factors). *Nevertheless, IL-10 expression, as a potent anti-inflammatory factor, potentially was upregulated via the lowest dose (0.05 %w/v) of gingerol compared to the control group (P < 0.01), which is explained in* Fig. 10*,* Table 7*.*

**Table 7:** ANOVA table for all immunological and biochemical parametric data with gingerol.

	groups	F* (DFn, DFd)	Means ± SD	P-value	Type of ANOVA test
IL-6		67.21 (5.000,18.17)		*P < 0.001*	Brown-Forsythe- Dunnett's-T3
	Sham		54.6 ± 10.66		
	Control		249.4 ± 28.24	−	
	0.05 %w/v		79.48 ± 18.73	*P < 0.001*	
	0.1 %w/v		161 ± 25.84	*P < 0.001*	
	0.3 %w/v		227.1 ± 42.33	Not	
	1 %w/v		252.4 ± 18.54	Not	
IL-10		1.961 (5, 30)		*P < 0.001*	Ordinary one-way ANOVA
	Sham		19.47 ± 5.507	*P < 0.001*	
	Control		51.25 ± 9.858	−	
	0.05 %w/v		66.85 ± 7.894	*P < 0.05*	
	0.1 %w/v		60.93 ± 11.06	Not	
	0.3 %w/v		47.08 ± 4.531	Not	
	1 %w/v		51.40 ± 10.16	Not	
TNF-α		141.5 (5.000,15.91)		*P < 0.001*	Brown-Forsythe- Dunnett's-T3
	Sham		50.18 ± 8.075	*P < 0.0001*	
	Control		315.7 ± 13.49	−	
	0.05 %w/v		104.8 ± 16.89	*P < 0.0001*	
	0.1 %w/v		213.2 ± 41.48	*P < 0.005*	
	0.3 %w/v		313.8 ± 25.08	Not	
	1 %w/v		313.9 ± 25.02	Not	
TGF-β1		0.9778 (5, 30)		*P < 0.0001*	Ordinary one-way ANOVA
	Sham		26.03 ± 5.255	*P < 0.0001*	
	Control		95.11 ± 11.47	−	
	0.05 %w/v		75.48 ± 9.996	*P < 0.05*	
	0.1 %w/v		83.60 ± 9.885	Not	
	0.3 %w/v		95.77 ± 7.299	Not	
	1 %w/v		102.4 ± 8.751	Not	
VEGF		0.8667 (5, 30)		*P < 0.001*	Ordinary one-way ANOVA
	Sham		31.8 ± 10.76	*P < 0.0001*	
	Control		111.2 ± 20.92	−	
	0.05 %w/v		62.59 ± 11.83	*P < 0.005*	
	0.1 %w/v		81.03 ± 11.44	*P < 0.05*	
	0.3 %w/v		104.6 ± 15.85	Not	
	1 %w/v		115.9 ± 13.38	Not	

Dfn: the degree of freedom for the numerator of the F ratio, DFd: degrees of freedom denominator, IL: Interleukin, TNF-α: tumor necrosis factor alpha, VEGF: vascular endothelial growth factor, TGF-β1: transforming growth factor beta 1. *P*-value is shown compared with control groups.

#### 3.5.2 Effects of gingerol on angiogenesis and fibrosis factors

PA induction made over-expression effects on VEGF and TGF-β cytokines that are important angiogenesis and fibrosis factors compared to the normal group (without PA induction) *(P < 0.0001*). Finally, the examination revealed that gingerol (0.05 and 0.1 %w/v) suppressed the angiogenesis via downregulation of the VEGF factor *(P < 0.0001, P < 0.001*). Surprisingly that, gingerol (0.05 and 0.1 % w/v) could inhibit the fibrosis by down-expression of TGF-β compared to the control group (P < 0.01 and P < 0.05, Fig. 10, Table 7).

## 4 Discussion

PA formation, a potential adverse event after post-operation, can reduce the patient's life quality (Liao et al., 2023). Our study was the first investigation of gingerol and ginger extract on PA in terms of basic science and clinical medicine. Various drugs and compounds, such as pharmacological, herbal compounds, and mechanical barriers, are systematically/locally investigated for PA prevention/inhibition (Abdullah and SAYDAM 2021). However, despite effective results, none of these have been accepted for standard therapeutics (Krämer et al., 2021). Considering the anti-inflammatory and anti-fibrotic efficacy of ginger and gingerol, they may be introduced as an herbal medicine for PA treatment via downregulation of IL-6, TNF-α, IL-10, VEGF, and TGF-β. In the present study, LC-MS was established for the simultaneous separation and identification of several chemical groups of ginger extract, including the gingerols, methylgingerols, gingerol acetates, shogaols, paradols, gingerdiols, mono- and diacetyl gingerdiols, and dehydrogingerdiones.

The current investigation reported many substantial events underlying the potential effects of ginger and gingerol on PA development. Our results also revealed a significant elevation in the level of the proinflammatory cytokines IL-6 and TNF-α and the level of angiogenic factors VEGF and TGF-β at the injury site after induction of post-operation PA. We found that ginger and its main ingredient, gingerol, improved wound healing, and their lower doses suppressed PA development. It is worth mentioning that over-expression of IL-6 and TNF-α rapidly induced post-operation PA, and VEGF and TGF-β elevation rapidly progressed the PA. The investigation of Yugang Sun et al. revealed that gingerol downregulated the 7,12-dimethylbenz[a]anthracene-induced overexpression of iNOS, IL-1β, IL-6, COX-2, and TNF-α as well as cell proliferation markers such as cyclin D1, proliferating cell nuclear antigen in hamster buccal pouch carcinogenesis models (Sun et al., 2021). Also, many other investigations of ginger and gingerol confirm our experiment results (Zehsaz et al., 2014, Aryaeian et al., 2019). Ultimately, the administration of ginger and gingerol prevented adhesion formation by reducing such cytokines.

Following PA, neutrophils, as dominant immune cells, shift to mostly macrophages in the injury site during 24 h post-injury. It is worth noting that the macrophages in the damaged area after the post-operation are different from resident macrophages. Post-operative macrophages can release plasminogen activator inhibitor (PAI), plasminogen activator (PA), lipoxygenase and COX metabolites, leukotriene B4, and prostaglandin E2, collagenase, elastase, interleukins-1, −6, TNF-α (Kharaziha et al., 2021, Raziyeva et al., 2021, Soltany, 2021). Particularly peritoneal macrophages have been indicated to be the main key in stimulating immune response to form adhesion. In fact, this is attributed to chemokine ligand 1 (CCL1) and Chemokine receptor 8 (CCR8). Inhibition of CCL1/CCR8 could also ameliorate PA in tissue injury (Fatehi Hassanabad et al., 2021, Soltany, 2021). Fibroblasts also play a crucial role in adhesion maturation during the second week of PA induction. Finally, connective tissue and vascular structures begin to develop on it (Fatehi Hassanabad et al., 2021, Gomes et al., 2021). Within a few days of development, the development of peritoneal adhesion becomes clearly visible (Gomes et al., 2021). TGF-β also potentially affects the production of fibroblasts without any exclusive influence (Frangogiannis, 2020). Degranulated mast cells may not be directly secrete VEGF locally, but they can stimulate the production of cytokines that lead to the infiltration of inflammatory cells to the sites affected by these cytokines (Mukai et al., 2018). Some researchers suggested that IL-17 and IL-6 have substantial roles in maintaining adhesions and chronic inflammation (Passos et al., 2010, Hu et al., 2021). TGF-β is the principal profibrotic mediator of the PA (Hu et al., 2018). In an animal model, TGF-β application to surgical adhesions induces PA exacerbation compared with the control group (Rahmanian-Devin et al., 2021). Moreover, PA formation can be decreased following exposure to TGF-β antibodies (Hu et al., 2021). As an intense angiogenic factor, VEGF directly contributes to early inflammatory activity and wound healing via intervention in fibroblast activity (Greaves et al., 2013). An *in-vivo* investigation proved that VEGF antibody potentially reduced PA (Bekes et al., 2016). Examination of post-surgical patients also revealed a major role of IL-6, −10 in the regulation of immune response to PA-associated inflammation (Jaafari et al., 2021). As IL-6 is an anti/pro-inflammatory cytokine that binds to its receptors (sIL-6R), it alters neutrophils to invading forms. Moreover, it influences the leukocyte recruitment into an influx of sustained mononuclear leukocytes in acute inflammation (Narazaki and Kishimoto, 2018). According to study of Jaafari et al., administration of *Portulaca oleracea* (for seven days) suppressed PA by upregulation of IL-10 and reduction of IL-6 (Jaafari et al., 2021). It has been reported that TNF-α can upregulate the expression of interleukin from mesothelial cells (Li et al., 2020). The range of TNF-α in serum and peritoneal fluid have associated with adhesion intensity in rats (Tang et al., 2020).

In this study, we focused on the ameliorative effects of gingerol and ginger extract on the peritoneum-secreted-related cytokines (IL-6, IL-10, VEGF, TNF-α, and TGF-β) following PA induction. Besides, ginger and gingerol downregulated the IL-6 expression against PA formation and reduced TNF-α induction. Their therapeutic effect on PA formation was also attributed to a significant reduction in the inflammatory cytokines, fibrosis, and angiogenesis biomarkers. However, only further experimentation can affirm the results of observational studies, and clinical trials prove that a new achievement is safe and effective.

## 5 Conclusion

Post-surgical PA illustrates a magnitude and fundamental global problem. Morbidity resulting from this disaster following peritoneal operation may be a life-threatening incident and a considerable burden on the patient. Our further knowledge of the multifactorial disorders of PA pathogenesis and our greater understanding of the cellular and molecular mediators' mechanisms will hopefully lead to the emergence of a preventive/therapeutic approach.

**Ethical approval**

The ethical committee accepted all procedures relating to animals based on the guidelines of animal experiments at the Mashhad University of Medical Sciences, Mashhad, Iran (20–10-2020, ethical approval code: IR.MUMS.MEDICAL.REC.1399.486).

**Authors' contributions**

R.Y., V.BR., SA.M, A.Y., M.I., M.H., and VR.A. wrote the first draft of the manuscript. All authors contributed to writing the project and read and approved the final manuscript submission. This study has been done by the authors mentioned in this article, and the authors will bear all responsibilities related to the contents of this article.

The authors are thankful to 10.13039/501100004748Mashhad University of Medical Sciences, Iran, for financial support (Approval date: 20-10-2020, ethical approval code: IR.MUMS.MEDICAL.REC.1399.486, approval ID: 990614, approval date: 25-11-2020).

## Declaration of competing interest

The authors declare that they have no known competing financial interests or personal relationships that could have appeared to influence the work reported in this paper.
